# The application of self‐limiting transgenic insects in managing resistance in experimental metapopulations

**DOI:** 10.1111/1365-2664.13298

**Published:** 2018-11-24

**Authors:** Liqin Zhou, Nina Alphey, Adam S. Walker, Laura M. Travers, Neil I. Morrison, Michael B. Bonsall, Ben Raymond

**Affiliations:** ^1^ Department of Life Sciences Imperial College London Ascot UK; ^2^ Department of Biosciences College of Life and Environmental Sciences University of Exeter Penryn UK; ^3^ Department of Zoology Mathematical Ecology Research Group University of Oxford Oxford UK; ^4^ The Pirbright Institute Surrey UK; ^5^ Oxitec Ltd Abingdon UK

**Keywords:** Cry1Ac resistance, diamondback moth, high‐dose/refuge strategy, metapopulation, population structure, resistance management, self‐limiting insects, transgenic insects

## Abstract

1. The mass release of transgenic insects carrying female lethal self‐limiting genes can reduce pest insect populations. Substantial releases are also a novel resistance management tool, since wild type alleles conferring susceptibility to pesticides can dilute resistance alleles in target populations. However, a potential barrier is the need for large‐scale area‐wide releases. Here, we address whether localized releases of transgenic insects could provide an alternative means of population suppression and resistance management, without serious loss of efficacy.
2. We used experimental mesocosms constituting insect metapopulations to explore the evolution of resistance to the *Bacillus thuringiensis* toxin Cry1Ac in a high‐dose/refugia landscape in the insect *Plutella xylostella*. We ran two selection experiments, the first compared the efficacy of “everywhere” releases and negative controls to a spatially density‐dependent or “whack‐a‐mole” strategy that concentrated release of transgenic insects in subpopulations with elevated resistance. The second experiment tested the relative efficacy of whack‐a‐mole and everywhere releases under spatially homogenous and heterogeneous selection pressure.
3. The whack‐a‐mole releases were less effective than everywhere releases in terms of slowing the evolution of resistance, which, in the first experiment, largely prevented the evolution of resistance. In contrast to predictions, heterogeneous whack‐a‐mole releases were no more effective under heterogeneous selection pressure. Heterogeneous selection pressure did, however, reduce total insect population sizes.
4. Whack‐a‐mole releases provided early population suppression, indistinguishable from homogeneous everywhere releases. However, insect population densities tracked the evolution of resistance in this system, as phenotypic resistance provides access to additional diet containing the toxin Cry1Ac. Thus, as resistance levels diverged between treatments, carrying capacities and population sizes increased under the whack‐a‐mole approach.
5. *Synthesis and applications*. Spatially density‐dependent releases of transgenic insects, particularly those targeting source populations at a landscape level, could suppress pest populations in the absence of blanket area‐wide releases. The benefits of self‐limiting transgenic insects were reduced in spatially localized releases, suggesting that they are not ideal for “spot” treatment of resistance problems. Nevertheless, spatially homogeneous or heterogeneous releases could be used to support other resistance management interventions.

## Abstract 2

Spatially density‐dependent releases of transgenic insects, particularly those targeting source populations at a landscape level, could suppress pest populations in the absence of blanket area‐wide releases. The benefits of self‐limiting transgenic insects were reduced in spatially localized releases, suggesting that they are not ideal for “spot” treatment of resistance problems. Nevertheless, spatially homogeneous or heterogeneous releases could be used to support other resistance management interventions.

## 1 INTRODUCTION

A range of pest management approaches, including chemical, biological, and cultural control can impose strong selection on the evolution of resistance (Onstad, 2013). Historically, there are very few strategies that have the potential to slow the evolution of resistance. One of the most effective is to reduce selection pressure through integrated pest management (IPM) and to apply pesticides only when strictly necessary (Forrester, Cahill, Bird, & Layland, 1993). However, successful IPM approaches depend on substantial research and require growers who are willing and able to apply knowledge‐intensive management strategies (Lacey & Shapiro‐Ilan, 2008). Other resistance management principles are well‐established and typically rely on access to two or more effective pesticides (Comins, 1977; Georghiou, Lagunes, & Baker, 1983; Georghiou & Taylor, 1977; Mani & Wood, 1984). These can be used in resistance management by applying selection pressure heterogeneously in time or space (rotations or mosaics) or from multiple active ingredients simultaneously in mixtures (Rex‐Consortium 2013; Roush, 1998).

Plant biotechnology, incorporating insecticidal toxins from *Bacillus thuringiensis* (*Bt*) into a range of crops, has changed the landscape for resistance management. With some important exceptions, doses of transgene‐encoded toxins are often high enough to ensure high levels of mortality and recessive resistance (Tabashnik, Gould, & Carriere, 2004). These high doses are particularly effective when used in conjunction with toxin‐free refugia, in the “high‐dose/refuge strategy.” When the inheritance of resistance is recessive, only homozygous‐resistant individuals (RR genotype) survive on transgenic crops. Another portion of the pest population is maintained in nearby refuges of non‐*Bt* host plants, providing a reservoir of susceptible alleles (from RS and SS genotypes, which survive in the refuge but not on the transgenic plants). If the resistance allele frequency is low, homozygous‐resistant pests surviving on *Bt* crops will be relatively rare, while susceptible pests will be abundant and available to mate with resistant individuals. Most progeny from such matings will be heterozygous for resistance alleles and phenotypically susceptible to high‐dose *Bt* crops, thereby hindering the evolution of resistance. Theoretical models and empirical observations have shown that the high‐dose/refuge strategy can effectively delay the development of resistance when resistance is recessive and when mating and oviposition are random (Alphey, Coleman, Bonsall, & Alphey, 2008; Alstad & Andow, 1995; Caprio, Faver, & Hankins, 2004; Gould, 1998; Gryspeirt & Gregoire, 2012; Huang, Andow, & Buschman, 2011; Hutchison et al., 2010; Téllez‐Rodríguez et al., 2014; Tyutyunov, Zhadanovskaya, Bourguet, & Arditi, 2008).

One recent development in insect genetic engineering has opened up a novel resistance management mode: the mass release of fertile, transgenic self‐limiting insects. Self‐limiting transgenic insects carry a dominant, repressible, lethal gene that can be sex‐specific in action (Thomas, Donnelly, Wood, & Alphey, 2000). In a strategy similar to sterile insect technique programmes, releasing large numbers of transgenic males can reduce target populations, as no viable offspring arise from mating of wild females and transgenic males (Alphey, Bonsall, & Alphey, 2009; Alphey, Coleman, Donnelly, & Alphey, 2007; Gentile, Rund, & Madey, 2015; Thomas et al., 2000). The term “self‐limiting” arises because these transgenes are designed to reduce insect fitness and will decline in frequency post‐release (Gould, Huang, Legros, & Lloyd, 2008).

If the only purpose of mass insect release was resistance management, fully fertile susceptible insects would more effective than those carrying lethal transgenes, but with obvious negative consequences for population size. The crucial feature of mass releasing self‐limiting transgenic males is that they can suppress pest population sizes and also affect the genetic make‐up of pest populations, if lethality is targeted only at females via engineered sex‐specific constructs. Alleles conferring susceptibility to insecticides carried by the released transgenic insects can then be introgressed into the target population through the male line. Deterministic models show that this technology can be a valuable tool in slowing the evolution of resistance (Alphey et al., 2007, 2009). Moreover, experiments with caged insects showed that this approach can slow the evolution of resistance to *Bt* in insects feeding on transgenic crucifers (Harvey‐Samuel et al., 2015). Also, resistance management with transgenic self‐limiting insects is compatible with other modes of resistance management (such as the high‐dose/refuge strategy) and can be deployed to complement them when a single strategy is insufficient to prevent resistance evolution (Zhou, Alphey, Walker, Travers, Hasan, et al., 2018).

Many applications of transgenic self‐limiting insects are currently envisaged as area‐wide management control techniques, in common with sterile insect release programmes (Carvalho et al., 2015; Thomas et al., 2000). Scale of operation can be one drawback to this type of pest management strategy and few are currently deployed by single grower or grower organizations (Winston, 1997). Area‐wide regimes require significant investment, and even eradication regimes can incur a commitment to long‐term spending even when pest densities are low or undetectable (Dyck, Hendrichs, & Robinson, 2005). Population suppression via sterile or transgenic insects is expected to be most effective when rolled out with high release ratios over large areas (Dyck et al., 2005; Vreysen, Carpenter, & Marec, 2010). This is especially important when eradication is the main target, as reinvasion of insects from untreated areas can quickly undo the work of years of investment.

However, if the purpose of transgenic insect release is population suppression (not eradication) *and* resistance management, it may be possible to achieve management goals without such large‐scale and long‐term economic commitments. Arguably, there are benefits for short‐term release programmes. For instance, resistance management efficacy is strongly dependent on the initial frequency of resistance alleles. This is particularly true for deployment of *Bt* toxins in the high‐dose refuge strategy (Alstad & Andow, 1995; Caprio et al., 2004; Gould, 1998). Increased levels of resistance can reach a tipping point when the frequency of resistance alleles is sufficient to ensure rapid increase in phenotypic resistance across that population (Roush, 1994). However, experimentally and theoretically, short‐term release of transgenic insects can reverse the evolution of resistance and potentially reduce resistance frequencies to a lower equilibrium that could be maintained by a high‐dose refuge strategy (Alphey et al., 2009; Zhou, Alphey, Walker, Travers, Hasan, et al., 2018). Thus, an intensive short‐term release programme could potentially reduce resistance frequencies to a level where they could be managed by other means.

**Figure 1:**
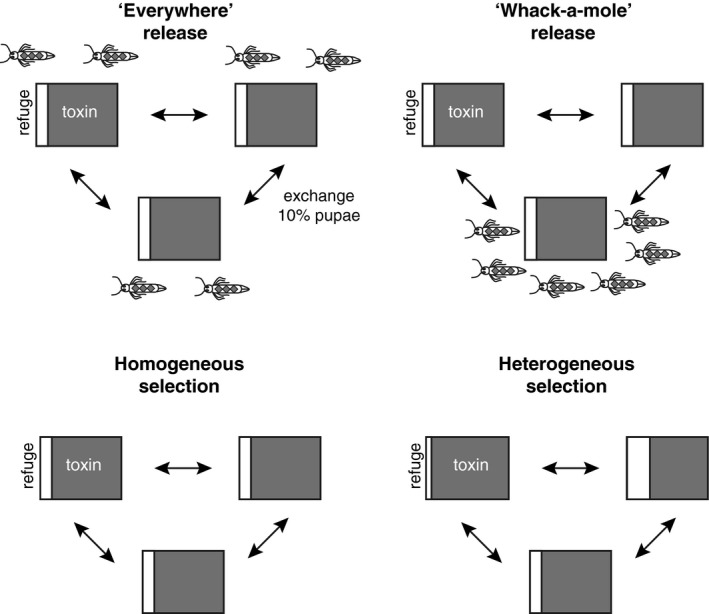
Design of selection experiments in this study. The first metapopulation experiment compared the application of a homogeneous everywhere transgenic release with a no‐release control and with a heterogeneous or “whack‐a‐mole” release. In the whack‐a‐mole strategy, self‐limiting insects were only deployed in the subpopulation in each network with the highest counts of survivors. The second metapopulation experiment examined the efficacy of everywhere and whack‐a‐mole control strategies when selection was homogeneous or heterogeneous. In the second experiment, varying the size of the toxin‐free refuge in different subpopulations imposed heterogeneity in selection pressure

Heterogeneity in space leads to differences in the risk of resistance evolution across the landscape. Population subdivision or structure is expected to lead to an increase in homozygosity, with resistance alleles concentrated in particular patches; this should accelerate the evolution of resistance (Caprio & Hoy, 1994). In addition to drift effects, variation in farming practice and in adherence to resistance management regimes could create heterogeneous selection pressure. Within a network of connected subpopulations, local populations with high levels of resistance are expected to have increased population size (Farias, Horikoshi, Santos, & Omoto, 2014; Gassmann, Petzold‐Maxwell, Keweshan, & Dunbar, 2011; Tabashnik, Van Rensburg, & Carrière, 2009) and so could act as sources of homozygous‐resistant individuals. Moreover, there may be benefits to focussing transgenic insect releases in particular subpopulations, to increase local release ratios of transgenic to wild males in areas most susceptible to resistance evolution. While there might be significant cost savings for less widespread release, the relative efficacy of focussed spatially heterogeneous transgenic insect releases has not been investigated experimentally. Here, our work had two aims. First, we assessed whether focussed spatially heterogeneous release of insects could have benefits for population suppression and resistance management, this we have termed the “whack‐a‐mole” approach, in reference to the popular game in which the local appearance of moles is combatted by localized application of a hammer (Figure 1). Second, we tested the hypothesis that spatially heterogeneous insect release would be more beneficial when selection for resistance was also spatially heterogeneous.

Building on our previous work (Zhou, Alphey, Walker, Travers, Hasan, et al., 2018), we investigate how spatially heterogeneous release of self‐limiting transgenic insects and the high‐dose/refuge strategy can control populations and mitigate the evolution of resistance in model experimental systems using the diamondback moth (DBM), *Plutella xylostella*. DBM is a widespread pest of cruciferous crops. Globally, DBM imposes management costs of US$1.3 billion–US$2.3 billion, and causes yield losses estimated at US$2.7 billion per annum (Furlong, Wright, & Dosdall, 2013; Zalucki et al., 2012). Control failure of DBM is a major concern in agriculture, as this species has developed resistance to almost every insecticide applied in the field as well as to microbial *Bt* sprays (Sarfraz & Keddie, 2005; Tabashnik, 1994). DBM is also a well‐established model for evaluating novel resistance management strategies (Raymond, Sayyed, Hails, & Wright, 2007; Zhao et al., 2005).

## 2 MATERIALS AND METHODS

### 2.1 Experimental conditions and insect populations

All insect populations were reared at 25°C (±1°C) and 45% (±5%) relative humidity, with a 12:12 light/dark cycle. DBM rearing procedures followed published protocols (Martins et al., 2012). Construction of the self‐limiting DBM (OX4319L, Oxitec Ltd) has been described previously (Jin et al., 2013). In brief, the self‐limiting system was implemented in our *Bt*‐susceptible line using sequences from the self‐limiting gene derived from the *doublesex* (*dsx*) gene of pink bollworm (Jin et al., 2013). Sex‐alternate splicing of this *dsx* sequence allows the development of a female‐specific lethal genetic system that is repressible by provision of tetracycline, or suitable analogues, in the larval feed (Jin et al., 2013). The OX4319L moths are denoted as genotype LL, where “L” represents the OX4319L construct insertion (Jin et al., 2013) and are all homozygous‐susceptible to Cry1Ac toxin (genotype SS).

Exogenous *B. thuringiensis* Cry1Ac was purified from *Escherichia coli* JM109 cells carrying the plasmid pGem1Ac, following published protocols (Cornforth, Matthews, Brown, & Raymond, 2015). The purified Cry1Ac toxin was incorporated into artificial diet (F9221B, Frontier Agricultural Sciences) to make toxin diet, at doses (0.5 μg/ml) sufficient to cause near‐recessive resistance (Zhou, Alphey, Walker, Travers, Hasan, et al., 2018). The construction of Cry1Ac‐resistant and susceptible DBM populations with similar genetic background (VB‐R and VB‐S respectively) has been described previously (Zhou, Alphey, Walker, Travers, Hasan, et al., 2018). Both VB‐R and VB‐S populations were non‐transgenic (ww genotype, where “w” represents wild type absence of the “L” construct).

### 2.2 Metapopulation experiment 1—Spatially homogeneous and heterogeneous release

Prior to selection experiments, we established a DBM population with a low initial frequency of resistance (“R” alleles) using mass crosses of homozygous‐susceptible VB‐S and resistant VB‐R populations. In the F1 generation, resistance allele frequency (7.5%) was confirmed by PCR and insect population size was increased to produce pupae for experiments. In the selection experiment, each replicate metapopulation consisted of three subpopulations that were established with 200 pupae (Figure 1). Transgenic releases of LL males (genotype LLSS) were timed to coincide with emergence of wild type counterparts. Eggs were collected twice per generation; 10% of the eggs were placed onto toxin‐free “refuge” diet and 90% of eggs on Cry1Ac toxin diet. After growth on respective diets and before the release treatment, pupae at different stages were pooled together into one cage per subpopulation. Dispersal between subpopulations within a replicate was imposed before mating: 10% of pupae in each subpopulation were shared between the two other subpopulations in each replicate (Figure 1).

This first selection experiment used three release treatments: (a) controls had no release of transgenic insects, (b) the “everywhere” release was a homogeneous strategy with transgenic male pupae released into all subpopulations at a ratio of four transgenic males to one wt male, (c) a treatment we termed “whack‐a‐mole,” was a spatially heterogeneous release in which we simulated a strategy designed to target subpopulations at greatest risk of evolving resistance, by releasing transgenic males at a 12:1 ratio into one subpopulation in each replicate with the highest number of survivors on toxin diet (Figure 1). All treatments were replicated three times, and each replicate consisted of three subpopulations. Note that the release ratio was three times higher (12:1 cf 4:1) where releasing into one‐third of the metapopulation, so that total numbers released were standardized across release treatments. In generation 0 (no releases), resistance levels were recorded prior to toxin selection, and pupal densities recorded after toxin selection. Thereafter, we monitored resistance in eggs laid after release of transgenic males, and prior to toxin selection, for three subsequent generations. Population sizes (number of pupae) emerging from toxin‐free diet (refugia) and toxin diet were recorded throughout the experiments.

We monitored evolving levels of Cry1Ac resistance in two ways. First, we used the survival of insects on toxin diet in experimental cages as a measure of resistance. All subpopulations were initiated with a 9:1 ratio of eggs on toxin and toxin‐free diet respectively, thus the proportion of insects surviving on toxin indicates the dynamics of phenotypic resistance within each cage. If survival rates on toxin and refuge diet are similar, indicating full resistance, 90% of total survivors in each cage will emerge from the toxin section. In contrast, in a fully susceptible population, there will be no survivors on the toxin section in each experimental subpopulation, and all survivors will emerge from the refuge. The proportional measure pupae survivors on toxin diet/total survivors in subpopulation, we have termed “Toxin survivors in cage.”

Second, we collected 10% of the eggs from each subpopulation (pooling for each replicate) and reared these separately in order to conduct bioassays of phenotypic resistance (genotype RR) under controlled conditions. These eggs were sampled prior to dividing eggs into toxin and refuge diet. Larvae for bioassays (*N* = 100 per replicate) were reared until 3rd instar and fed on diet containing a dose of 0.5 μg/ml Cry1Ac to assess susceptibility. Note the bioassays from eggs indicate levels of resistance prior to transgenic insect release, while the “toxin survivors in cage” measure reflects the survival of larvae that are the progeny of local adults and the released transgenic males.

### 2.3 Metapopulation experiment 2—Homogeneous and heterogeneous selection pressure

The second selection experiment tested the hypothesis that heterogeneous release of transgenic insects (the “whack‐a‐mole” strategy), would have improved efficacy relative to when selection in the population itself was heterogeneous. The experimental set‐up and monitoring methods were as above. We ran a factorial experiment that varied release strategy (everywhere or whack‐a‐mole, as above) and selection pressure (homogeneous or heterogeneous). We imposed heterogeneous selection pressure by allocating different refugia sizes to each subpopulation within a replicate, assigning refugia of 5%, 10%, and 20% randomly each generation (Figure 1). Under homogeneous selection pressure, we used a 12% refuge in all subpopulations every generation (Figure 1) to provide the same overall mean refuge size in homogeneous and heterogeneous selection treatments. Bioassays were conducted as above, except that 150 third instar larvae were tested for phenotypic resistance.

### 2.4 Statistical analyses and experimental design

Statistical analysis was carried out in R (https://cran.r-project.org) using analysis of variance, generalized linear modelling, and mixed effect models. The numbers of pupal survivors from selection diet and refuge diet were analysed with normal errors after square root transformation. Proportional data were analysed with GLMMs with binomial errors in *lme4*: mixed model analyses used replicate (within generation) as a random effect, if subpopulation level data were used in analyses, this was nested within replicate. Statistical tests primarily used model simplification and Likelihood ratio tests and, where appropriate (for non‐nested models), the Akaike information criterion (AIC). Mixed model analytical results for binomial data (bioassays and toxin survivors) were confirmed using arc‐sine transformed proportions in *lmer* models—these gave qualitatively similar results (Supplementary Materials). All model assumptions were checked with graphical analysis of error distribution assumptions. Raw data for these experiments are available from Dryad (Zhou, Alphey, Walker, Travers, Morrison, et al., 2018).

## 3 RESULTS

### 3.1 Metapopulation experiment 1—Spatially homogeneous and heterogeneous release

**Figure 2:**
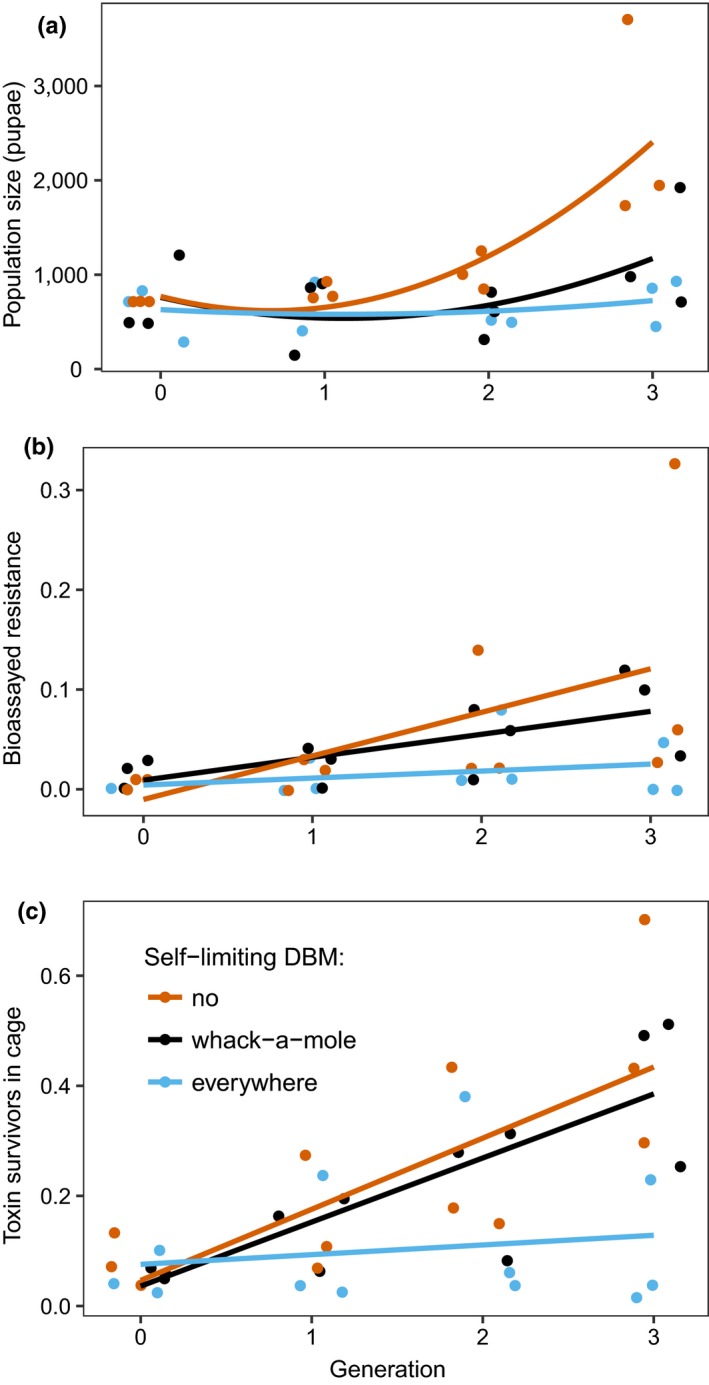
Efficacy of spatially homogeneous and heterogeneous release of transgenic self‐limiting insects in managing insect populations and evolution of resistance to the *Bt* toxin Cry1Ac. Here, selection on toxin resistance began in generation 0, but transgenic insect release started at the beginning of generation 1, as we required data on variation in local population size to deploy the whack‐a‐mole strategy. (a) Population size (sum of three subpopulations) over four generations in controls (orange), everywhere release (blue) and whack‐a‐mole (black) release. These data are total numbers of pupae surviving toxins and transgenes at the end of each generation. (b) Phenotypic resistance (proportion of homozygous‐resistant larvae) in larvae reared from eggs collected across each network (c) Proportion of survivors on toxin diet in experimental cages. Proportion of toxin survivors represents the homozygous‐resistant survivors (RR pupae) from Cry1Ac diet divided by the total pupal survivors pooled from both Cry1Ac diet and refuge diet in each cage population; data are means of three subpopulations; lines are fitted quadratic models. Experiments used a 10% refuge size

The application of self‐limiting transgenic insects successfully reduced insect populations in the “everywhere” and “whack‐a‐mole” treatments compared with controls. Population size grew rapidly in controls but only slowly in both transgenic release treatments (Figure 2a, treatment:generation^2^, Likelihood ratio test = 10.1, *df* = 2,11, *p* = 0.0064). Generation (as a linear term) did not significantly interact with treatment or have a strong main effect (generation × treatment; Likelihood ratio test = 1.06, *df* = 2, *p* = 0.69; generation main effect − Likelihood ratio test = 2.96, *df* = 1, *p* = 0.0852). Importantly, “everywhere” and “whack‐a‐mole” treatments were equally effective in terms of population suppression (factor level reduction − Likelihood ratio test = 1.97, *df* = 2, *p* = 0.37). The model with only two treatment levels (control and both transgenic release treatments combined) also had the lowest AIC (240.4 vs. 242.4). Since controls diverged from transgenic treatments as the experiment proceeded, arguably, we have limited power to distinguish everywhere and whack‐a‐mole interventions. Nevertheless, the raw data support the statistical inference, population sizes in the whack‐a‐mole approach tightly overlap the everywhere approach, with the exception of two subpopulations in generation 3 only (Figure S1).

Note that population increases in these experiments were linked to the evolution of resistance. At the subpopulation level, variation in total population size tended to increase in each cage only when the survivors on toxin diet began to exceed 40% of the total population (Figures S1 and S2). The proportion of insects in cages surviving on toxin was a significant predictor of population size (Likelihood ratio test = 34.1, *df* = 1, *p* < 0.0001). Note: this is calculated as the number of pupae surviving from toxin diet divided by the entire pupal population size, which will therefore vary from 0 (no phenotypic resistance) to a maximum of 0.9 (see methods above).

Bioassays of phenotypic resistance (sampled from eggs collected after transgenic release) showed that resistance gradually increased over the course of this experiment (Figure 2b; generation main effect − Likelihood ratio test = 6.24, *df* = 1, *p* = 0.012). However, bioassayed resistance increased slowly in all but one control replicate (in which resistance increased to >10%), and experimental treatments did not differ from each other in their rate of increase in resistance (treatment × generation interaction − Likelihood ratio test = 4.7, *df* = 1, *p* = 0.095). Nevertheless, the proportion of insects surviving on toxin diet in experimental cages can also be used to track levels of phenotypic resistance. This statistic reflects the proportion of resistant insects at the end of each generation, after selection from toxins and mortality transgenes had acted on larvae. These data showed that the everywhere release largely prevented the evolution of resistance (Figure 2c, treatment × generation interaction − Likelihood ratio test = 29.0, *df* = 2, *p* < 0.0001). However, in this scenario the whack‐a‐mole release and the control treatments were indistinguishable (factor level reduction − Likelihood ratio test = 0.6, *df* = 2, *p* = 0.74).

Although these experiments represent a short time series, we also investigated whether the dynamics in resistance (generation to generation variability, as opposed to overall trends) within subpopulations might be affected by release of transgenic insects. We tested for evidence of temporal autocorrelation in the proportion of survivors on toxin diet within mixed models fitted in *lme*. Analysis of the autocorrelative patterns shows a significant negative autocorrelation with lag 1 in the experiment as a whole (Figure S3). Exploring variation between treatments suggests this lag is largely driven by the release of transgenic insects. The biological interpretation is that the whack‐a‐mole treatment was performing as expected, in terms of locally suppressing evolution of resistance in the generation following peak abundance of resistant insects. Graphical inspection of detrended data supports this interpretation (Figure S3). While inclusion of temporal autocorrelative terms in models did not formally improve explanatory power, or decrease AIC, this may be because the quadratic term in the mixed models is already capturing substantial autocorrelation.

### 3.2 Metapopulation experiment 2—Homogeneous and heterogeneous selection pressure

**Figure 3:**
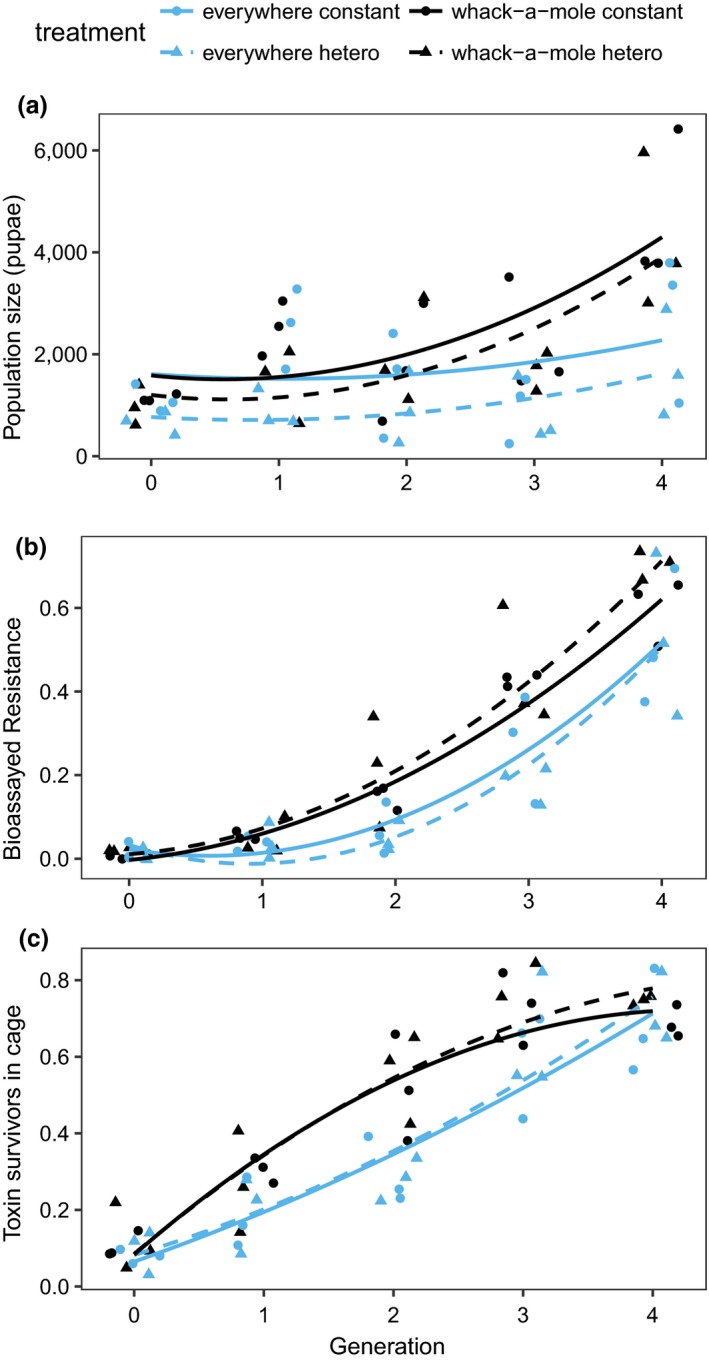
Efficacy of spatially homogeneous (everywhere) and heterogeneous (whack‐a‐mole) releases of transgenic self‐limiting insects in population networks under homogeneous (circles, solid lines) and heterogeneous selection pressure (triangles, dashed lines. a) Population size (sum of three subpopulations) over four generations in everywhere release and whack‐a‐mole release. Again these data are total numbers of pupae surviving at the end of each generation of selection. (b) Phenotypic resistance (proportion of homozygous‐resistant larvae) in larvae reared from eggs collected across each network (c) Proportion of survivors on toxin diet in experimental cages. Proportion of toxin survivors represent the ratio of homozygous‐resistant survivors (RR pupae) from Cry1Ac selection diet to total pupae survivors pooled from selection diet and refuge diet in each cage population; data are means of three subpopulations

In this experiment, we predicted that the whack‐a‐mole strategy might be more advantageous under heterogeneous selection pressure, that is, if there were subpopulations where we would expect rapid evolution of resistance (because more of the diet contained toxin and less was refuge). In terms of controlling population size, neither transgenic deployment method could prevent populations increasing in this experiment (quadratic generation term − Likelihood ratio test = 5.0, *df* = 1, *p* = 0.025; Figure 3a). Populations showed only modest increase in the first three generations, as we saw in the first selection experiment. Nevertheless, over the course of the whole experiment the everywhere release proved significantly better at slowing population growth (treatment × generation interaction − Likelihood ratio test = 5.42, *df* = 1, *p* = 0.02; Figure 3a). The nature of selection pressure (homogeneous vs. heterogeneous) did impact population size overall: populations were reduced under the heterogeneous selection regime (selection regime main effect − Likelihood ratio test = 4.37, *df* = 1, *p* = 0.037; Figure 3a); however, selection regime did not interact with release treatment (Likelihood ratio test = 0.77, *df* = 1, *p* = 0.38) or with generation (Likelihood ratio test = 0.015, *df* = 1, *p* = 0.90).

Release treatment also affected the rate of change of phenotypic resistance in bioassays (treatment × generation^2^ interaction − Likelihood ratio test = 8.12, *df* = 1, *p* = 0.0043; Figure 3b). But in contrast to our hypothesis, the selection regime did not affect the levels of resistance observed in bioassays (selection regime main effect − Likelihood ratio test = 0.022, *df* = 1, *p* = 0.88), nor did selection regime interact with release treatment (treatment × selection interaction Likelihood ratio test = 1.47, *df* = 1, *p* = 0.225) or generation (selection × generation^2^ interaction − Likelihood ratio test = 2.58, *df* = 1, *p* = 0.11). Similar patterns can be seen in the survival of insects on the toxin diet in experimental cages: resistance increases more quickly in the whack‐a‐mole release treatment (treatment × generation interaction − Likelihood ratio test = 12.8, *df* = 1, *p* = 0.00034, and treatment × generation^2^ interaction − Likelihood ratio test = 186.6, *df* = 1, *p* < 0.0001; Figure 3c). As with bioassays, the selection regime did not affect the dynamics of insect survival on toxin (interaction between selection and generation or treatment all *p* > 0.05; Figure 3c). Note that the proportion of toxin survivors in the whack‐a‐mole treatment is decelerating as it is approaching its maximum of 0.88, determined by the refuge size of 12%.

## 4 DISCUSSION

Release of self‐limiting insects carrying sex‐specific transgenes clearly has potential to reduce insect population sizes and the rate of evolution of resistance (Alphey et al., 2007, 2009; Harvey‐Samuel et al., 2015; Zhou, Alphey, Walker, Travers, Hasan, et al., 2018). Here, we extended this work to test whether spatially heterogeneous releases of transgenic insects (whack‐a‐mole strategies) could also provide a means of population suppression and resistance management, in comparison to homogeneous area‐wide management. The first metapopulation experiment demonstrated that focusing insect releases on subpopulations with the highest densities of resistant insects could provide population suppression relative to controls (no transgenic insects), and this was not significantly less effective than homogeneous area‐wide approaches.

This suggests that focusing transgenic population suppression on population sources, that is, subpopulations with net positive population growth (Pulliam, 1988; Pulliam & Danielson, 1991), rather than across the entire landscape, could be a viable strategy. Although random spatially restricted release is expected to less efficient (Legros et al., 2012). Targeting population sources with transgenic insects provides an additional landscape level of density‐dependent regulation; reducing the frequency of population source to sinks across the landscape is expected to lower pest densities and influence local extinctions (Pulliam, 1988; Pulliam & Danielson, 1991). Understanding how spatial structure and sink patches alter the emergence and management of resistance warrants further investigation (Caprio, 2001). A practical issue, of course, is whether source populations can be readily identified in the pest management landscape. In this experiment, source populations could be inferred from the presence of viable insects on toxin diet. In the field, this would be akin to observing significant damage on transgenic or sprayed crops. Nevertheless, this strategy need not be applied reactively. For many economically important pests, the climatic, altitudinal, seasonal, and/or landscape factors driving population growth are well‐known (Carrière et al., 2012; O'Rourke & Jones, 2011; Rand, Waters, Blodgett, Knodel, & Harris, 2014).

There was more of a mixed picture for the relative resistance management efficacy of homogeneous and heterogeneous insect release. In assessing levels of resistance using bioassays of eggs sampled immediately after release of transgenic insects, there was little to differentiate the everywhere and whack‐a‐mole release strategies. We did, however, see evidence of increased resistance within the whack‐a‐mole strategy in terms of number of survivors on toxin diet, while resistance was stabilized in the everywhere release strategy, in common with earlier work (Zhou, Alphey, Walker, Travers, Hasan, et al., 2018). These data are not inconsistent, since toxin selection occurs at the larval stage, the pattern of survival at the pupal stage reflects resistance after an additional round of selection. Thus, frequencies of resistance are likely to be different when we compare insects sampled before and after transgenic release of adult males.

Plant‐ or diet‐incorporated Cry toxins can substantially reduce insect population sizes in the field (Carrière et al., 2003; Hutchison et al., 2010; Wu, Lu, Feng, Jiang, & Zhao, 2008) and in experimental laboratory populations (Harvey‐Samuel et al., 2015; Zhao et al., 2005; Zhou, Alphey, Walker, Travers, Hasan, et al., 2018). The evolution of resistance will reduce the impact of toxin on population size, and increased pest population sizes are expected to lag behind the evolution of resistance, as we saw here. A gradual increase in the frequency of resistance alleles will eventually lead to increases in the numbers of resistant homozygotes that are sufficient to ensure the collapse of the high‐dose refuge strategy (Carrière & Tabashnik, 2001; Gould, 1998; Ives & Andow, 2002). It is likely therefore, that the slightly weaker resistance management in the whack‐a‐mole approach would lead to larger population sizes in the long run, which indeed happened when we extended the second metapopulation experiment to a fifth generation.

While we anticipated that the whack‐a‐mole release strategy might not be as fully effective as a homogeneous release programme, we hypothesized that any drawbacks might be reduced when selection for resistance was also heterogeneous. However, the data did not support this hypothesis. Heterogeneity in selection pressure tended to reduce population sizes, but had no impact on overall evolution of resistance and did not interact with selection treatment (Figure 3). Possibly the weaker effect of heterogeneity in selection pressure arose because experimental manipulation was masked by variation between subpopulations in all treatments (Figures S1 and S4). Although there was limited variation in resistance between subpopulations within replicates at the start of experiments, in some instances trajectories in resistance diverged quite quickly between subpopulations (Figures S2 and S5). Although not possible in this experiment, there is one possible real‐world compromise to the approaches tested here, this would be to locally increase release ratios in suspected source populations or hot‐spots of resistance. This might help reduce emigration of homozygous‐resistant insects but at the same time not undermine the efficacy of the area‐wide releases.

The data across both metapopulation experiments indicate that spatially heterogeneous release of insects is inferior to area‐wide releases for resistance management, at least under the conditions described here. One potential drawback of rotating transgenic release between subpopulations, not apparent at the start of this study, is that multiple generations of release tend to produce more robust population suppression (Zhou, Alphey, Walker, Travers, Hasan, et al., 2018). The theoretical explanation for this is that the self‐limiting alleles are inherited in the target population with the released homozygous males producing male progeny that carry one copy of the transgene. In subsequent generations, those heterozygous males can compete for mates with wild type males, in addition to newly released homozygous transgenic males. With continuous releases at the same site, the proportion of heterozygous males in the targeted population will increase, providing additional population suppression, while after releases have ceased the proportion of heterozygotes declines rapidly. Thus, moving release of transgenic insects between subpopulations could further reduce the efficacy of pest suppression. It is possible that targeted releases, which focus on fixed, problematic source populations, and which allow local build‐up of transgenes may be more effective than regularly changing release sites, as we did here.

Other parameters likely to affect the relative benefits of targeted release include the dispersal capabilities of targeted pests, the pattern of dispersal, as well as the timing of mating relative to dispersal. Clearly, if there is only limited dispersal between subpopulations, then the value of targeted release increases. In this experimental system, dispersal occurred before mating. The timing of dispersal in relation to mating can vary within and between insect Orders (Johnson, 1969). In general, if dispersal occurs *before* mating, then the effects of any local increases in homozygosity will be moderated, which should reduce the rate of evolution of resistance (Ives & Andow, 2002). If dispersal occurs *after* mating, then homozygous‐resistant eggs produced in subpopulations with high levels of resistance can effectively spread through the network. Since local, focused whack‐a‐mole strategies are expected to work more efficiently if problems are localized, it follows that spatially heterogeneous deployment of transgenic adults might be more effective when insects mate after dispersing.

Differences in resistance evolution between homogeneous and heterogeneous release treatments in these experiments must be put into context. Here, in order to detect differences between treatments, the experimental set‐up was constructed to generate rapid evolution of resistance: initial frequencies of resistance alleles are high (much greater than 1%); while resistance is very effective and imposes relatively minor fitness costs (Zhou, Alphey, Walker, Travers, Hasan, et al., 2018). In the first experiment, resistance steadily increased in one of the three homogenous release replicates, while all three replicates evolved resistance in the whack‐a‐mole strategy. In the second experiment, homogeneous release slowed evolution of resistance, but this was only one generation behind the whack‐a‐mole approach. In terms of the expected lifetime of many GM toxins, this is a relatively modest difference.

More dramatic difference in resistance evolution outcomes will come about if area‐wide release can facilitate stable and low levels of resistance across a wider range of parameter values (initial frequency of resistance, fitness costs, refugia area, etc.). This is a substantial theoretical challenge and beyond the scope of this paper. Nevertheless, experience with this experimental system suggests some valuable lessons. For instance, for the more ambitious management aims, such as reversing evolution of resistance in widely dispersing insects, which is difficult but theoretically and practically possible (Alphey et al., 2009; Zhou, Alphey, Walker, Travers, Hasan, et al., 2018), an area‐wide approach may be the only sensible option. On the other hand, transgenic insect release can support other, failing, resistance management strategies (Figure 2) (Zhou, Alphey, Walker, Travers, Hasan, et al., 2018). In many countries, resistance monitoring is mandated by regulations licensing *Bt* crops, and several countries have noted a gradual rise in resistance alleles that falls short of full field resistance and crop failure, but which may indicate incipient resistance (Downes, Parker, & Mahon, 2010; Tabashnik, Gassmann, Crowder, & Carriere, 2008, 2008; Zhang et al., 2011). If early warning systems are signalling only a gradual increase in resistance, this suggests that more modest intervention strategies (e.g., targeted transgenic releases) may be sufficient to stabilize evolutionary dynamics.

## AUTHORS’ CONTRIBUTIONS

B.R. conceived the study; while M.B.B., N.I.M., N.A. and L.Z. contributed to experimental design. L.Z., L.M.T. & A.S.W. carried out experiments; L.Z. and B.R. analysed data. B.R. wrote the first draft and all authors contributed to and approved the final version.

## Data Availability

Data available from the Dryad Digital Repository https://doi.org/10.5061/dryad.5cm7088 (Zhou, Alphey, Walker, Travers, Morrison, et al., 2018).
