# Supplementary material for: The application of self‐limiting transgenic insects in managing resistance in experimental metapopulations
Source: J Appl Ecol. 2018 Nov 24;56(3):688–98. doi: 10.1111/1365-2664.13298 (PMC6446822; doi:10.1111/1365-2664.13298)

Figure S1. Variation in population size at the sub-population level in meta-population experiment 1. Lines are fitted quadratic models for each treatment.

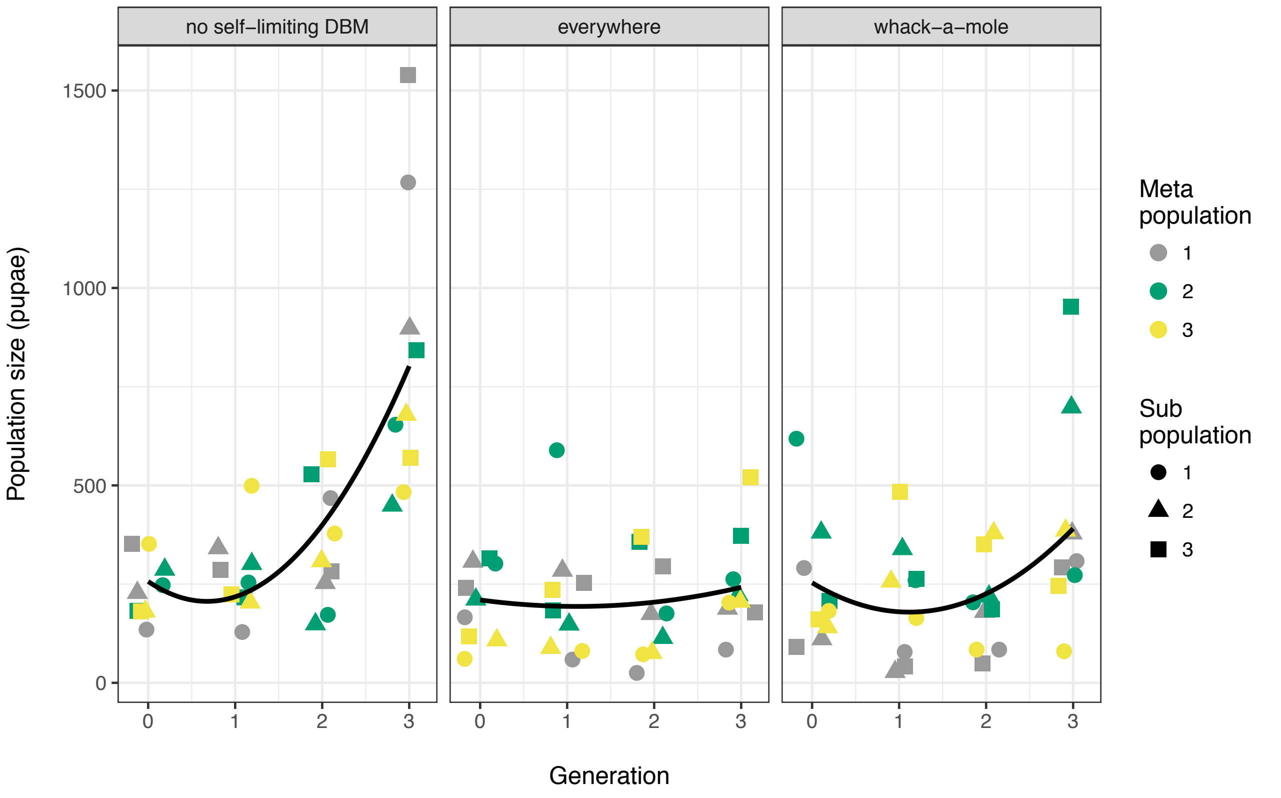

Figure S2. Variation in proportion of toxin survivors at the sub-population level in meta-population experiment 1. Lines are fitted quadratic models for each treatment.

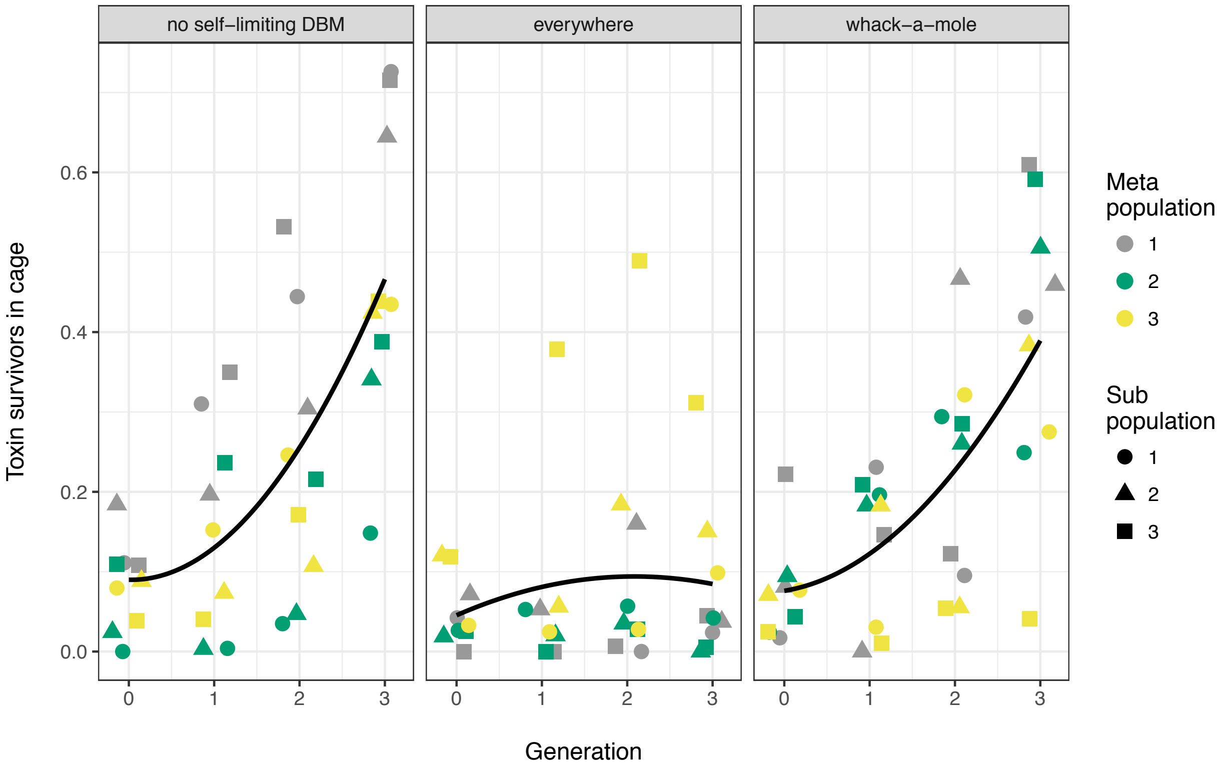

Figure S3. Autocorrelation analyses for mixed models of the proportion of toxin survivors (arc-sine transformed) in meta-population experiment 1. ((A) All treatments. ((B) Transgenic insect release treatments. (C) Control treatments. Blue dashed lines indicate statistically significant autocorrelation at $P = 0.05$. Statistical models (in *lme*) used generation as linear and quadratic terms and have subpopulation nested within replicate as random effects. (D) Data in the whack a mole treatment were de-trended by subtracting fitted model values from raw data.

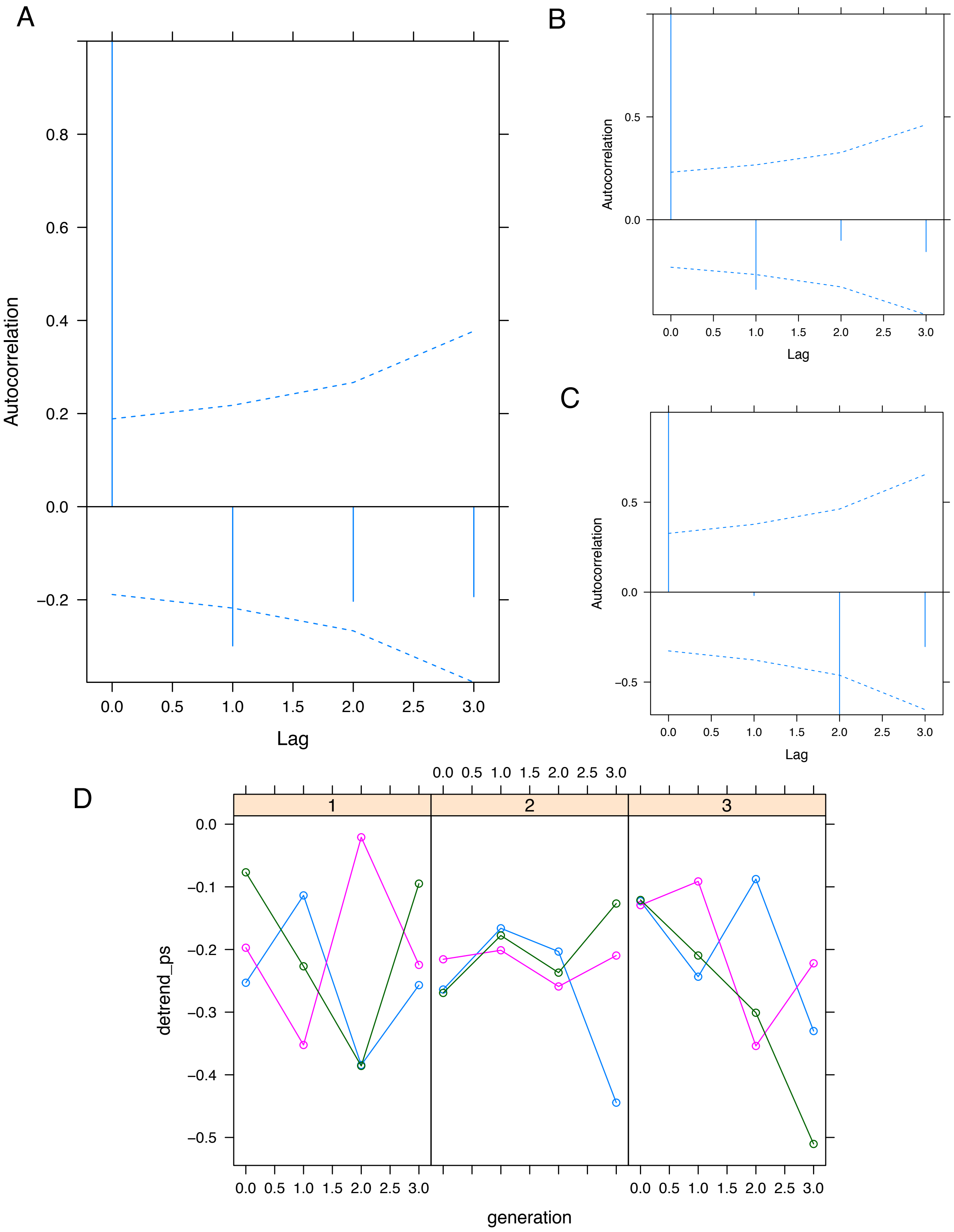

Figure S4 and S5. Variation in population size at the sub-population level in meta-population experiment 2. Lines are fitted quadratic models for each treatment.

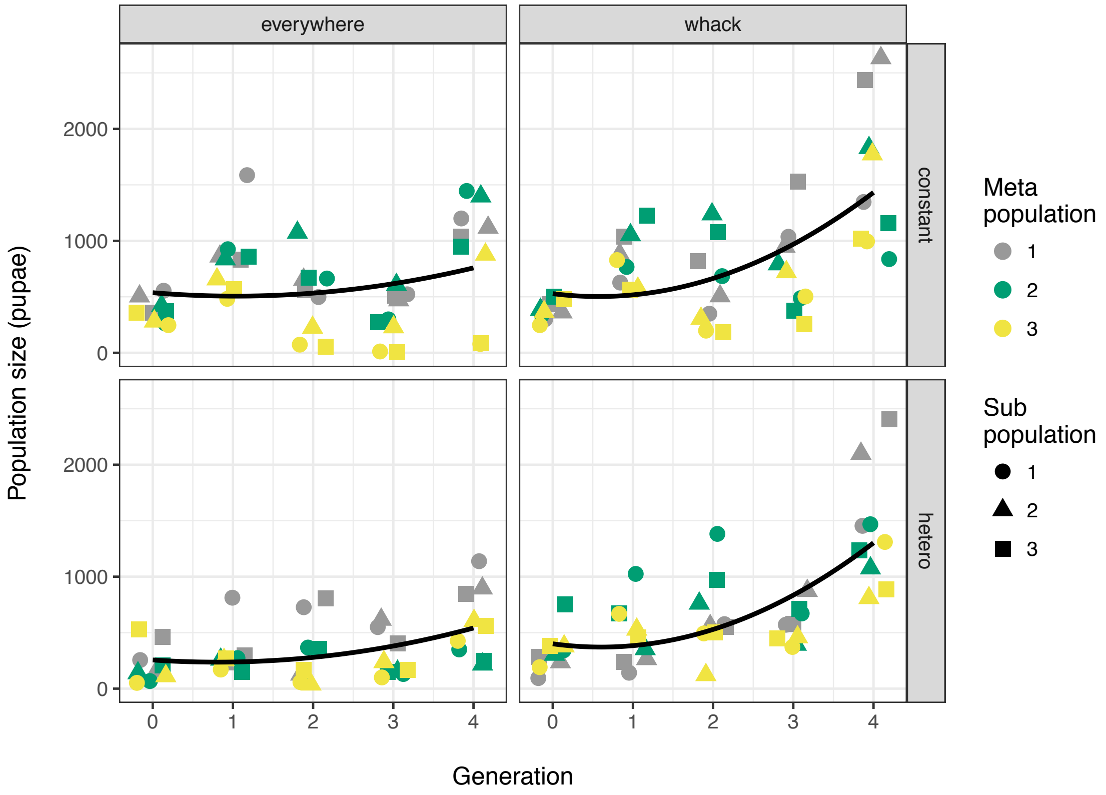

Figure S5. Variation in proportion of toxin survivors at the sub-population level in meta-population experiment 2. Lines are fitted quadratic models for each treatment.

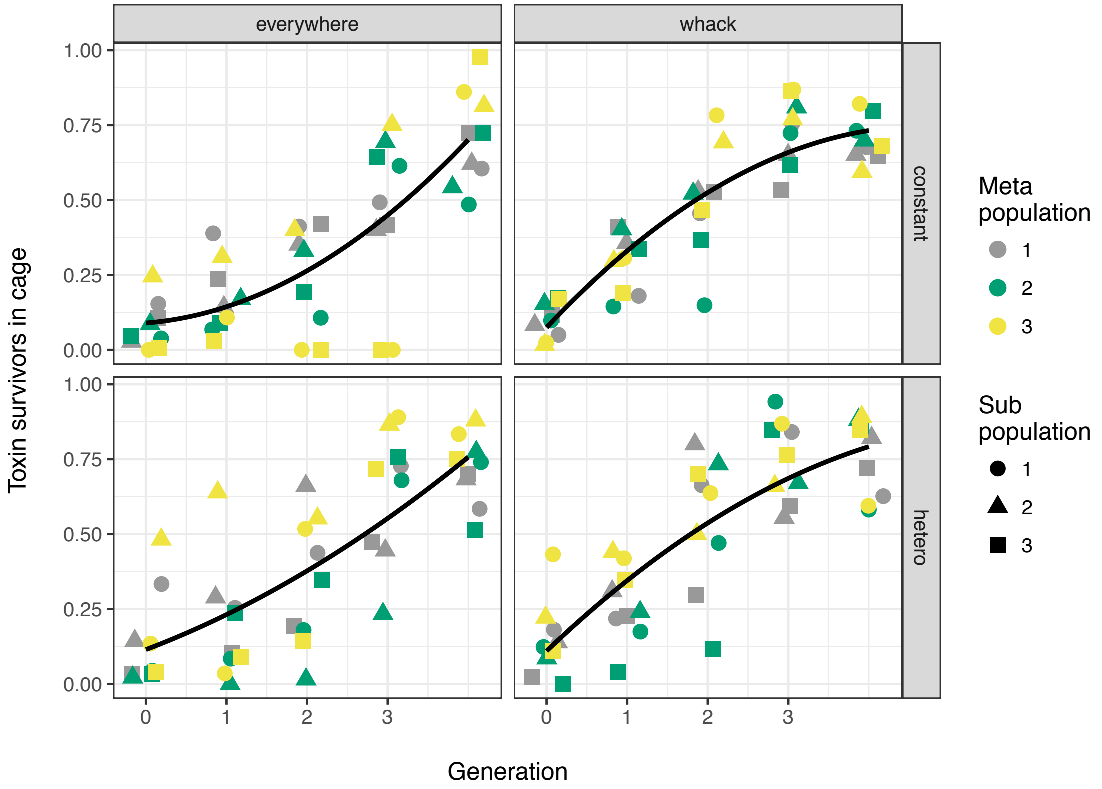

Supplement: Supplementary file 1 [file JPE-56-688-s001.pdf]
